# Features of ovarian Brenner tumors: Experience of a single tertiary center

**DOI:** 10.4274/tjod.98216

**Published:** 2017-06-15

**Authors:** Batuhan Turgay, Kazibe Koyuncu, Salih Taşkın, Uğur Fırat Ortaç

**Affiliations:** 1 Ankara University Faculty of Medicine, Department of Obstetrics and Gynecology, Ankara, Turkey

**Keywords:** Brenner tumor, ovarian neoplasm, Gynecologic Oncology

## Abstract

**Objective:**

Brenner tumors are rare neoplasms of the ovary. The aim of this study was to investigate the clinical features of Brenner tumors.

**Materials and Methods:**

The clinical features of 22 patients who were treated in Ankara University Faculty of Medicine Obstetrics and Gynecology Department between 2005 and 2015 were evaluated retrospectively from hospital medical records.

**Results:**

The patients were aged 34 to 79 years at the time of diagnosis and the mean age was 55.1 years. Two (9.1%) patients were pre-menopausal, five (22.7%) were peri-menopausal, and 25 (68.2%) patients were postmenopausal. One patient was pregnant. Twenty of the neoplasms were benign, one was malignant, and one was both malignant and benign. There was no recurrence in the malignant cases. Six (27.2%) patients had mixed tumors consisting of Brenner tumor and another ovarian pathology. Specifically, the other component of these tumors was mucinous cystadenoma in four patients, endometriosis externa in one patient, and high-grade serous papillary cyst adenocarcinoma in one patient.

**Conclusion:**

Brenner tumors are usually incidental benign pathologic findings of surgical procedures in postmenopausal women. They can be found with other ovarian pathologies such as mucinous ovarian tumors and can coexist with other female genital tumors. Further studies are needed to completely understand the clinical features of Brenner tumors.

## PRECIS:

We have investigated the clinical features of rare Brenner tumors with twenty two cases were operated in our clinic.

## INTRODUCTION

Brenner tumors are a relatively rare surface epithelial neoplasm of the ovary, accounting for 1.4-2.5% of all ovarian tumors. They are known as transitional cell tumors of the ovaries due to their histologic findings. Brenner tumors usually affect postmenopausal women, and most (99%) are benign^(1,2,3,4)^. They are usually unilateral; bilateral lesions are found in 5-14% of cases^(2,4,5)^. Although Brenner tumors are usually discovered incidentally, patients occasionally present with symptoms such as a palpable mass or pain^(6)^. Here, we present the clinical findings of Brenner tumors that were diagnosed and treated between 2005 and 2015 in Ankara University, Department of Obstetrics and Gynecology.

## MATERIALS AND METHODS

Patients with a histologic diagnosis of Brenner tumor of the ovary who underwent laparotomic or laparoscopic surgery for any reason between 2005 and 2015 were identified from the database of Ankara University’s Faculty of Medicine Department of Obstetrics and Gynecology. The records of 22 patients were diagnosed as having Brenner tumors were retrieved. Data on age, clinical findings, menopausal status, tumor size, surgical procedure, associated pathologic findings, side of the tumor, and clinical follow-up period were collected. Follow-up information was taken retrospectively from the medical records of patients’ last visits of our hospital. In this study, all data were assessed retrospectively and neither ethics committee approval nor patients’ informed consents were obtained. This study was reviewed by the appropriate ethics committee and was performed in accordance with the ethical standards described in an appropriate version of the 1975 Declaration of Helsinki, as revised in 2000.

### Statistical Analysis

Data analysis was performed using IBM SPSS Statistics 20.0 software (IBM Corporation Software Group, New York, United States of America).

## RESULTS

Twenty two cases of Brenner tumor were diagnosed over a ten-year period. The clinical characteristics are summarized in Table 1. The patients were aged 34 to 79 years at the time of diagnosis and the mean age was 55.1 years. Two (9.1%) patients were pre-menopausal, five (22.7%) were peri-menopausal, and 15 (68.2%) were postmenopausal. One patient was pregnant.

Eight (36.3%) patients were admitted to our department because of adnexal mass and their main symptom was abdominal pain. The other patients’ main symptoms were divergent. Seven (31.8%) patients presented with postmenopausal bleeding, two (9.1%) with menometrorrhagia, two (9.1%) with uterine descensus, one (4.55%) with ascites, and one (4.55%) with vaginal bleeding and a vaginal mass. Among the patients, only two had clinical symptoms caused by Brenner tumors and these were malignant.

The left ovary was involved in 12 cases, the right in 8 cases, and bilateral Brenner tumors existed in 2 cases (9.1%). The smallest tumor had a maximum diameter of 2 mm and the largest tumor a maximum diameter of 20 cm. Twenty of the neoplasms were benign, one was malignant, and one was both malignant and benign.

Five (22%) patients underwent surgery for endometrial malignancy and one for cervical malignancy. Nine patients presented with adnexal mass or ascites. Among these patients, the final pathology of two patients was benign pure Brenner tumor. Two cases (9.1%) were diagnosed as malignant Brenner tumor, and one as high-grade serous papillary cyst adenocarcinoma. Four cases (18.2%) were mixed tumors consisting of Brenner tumor and another ovarian pathology. Specifically, the other component of these tumors was mucinous cystadenoma. One patient was admitted with uterine descensus and their final pathology was coexistence of endometriosis externa and Brenner tumor. One cesarean section was performed for the previous cesarean delivery indication and a benign Brenner tumor was diagnosed incidentally.

Case 10 had a bilateral malignant Brenner tumor (stage III C) and she underwent total abdominal hysterectomy with bilateral salpingo-oopherectomy, ommentectomy, bilateral pelvic paraaortic lymphadenectomy, splenectomy, and appendectomy. The preoperative CA-125 was 51 U/mL. The patient received six cycles of carboplatin-paclitaxel chemotherapy and has been in follow-up for 2 years with no recurrence. The final pathology of case 11 was grade 2, stage III C malignant Brenner tumor in the right ovary, and a benign tumor in the left ovary. The patient received the same chemotherapy protocol as case 10 and there was no recurrence after surgery during 18 months of follow-up.

Fifteen patients’ tumor marker CA-125 levels were known before surgery. Levels of CA-125 were high in only two patients; they were normal in the remaining patients (CA-125 cut-off level 35 IU/mL).

## DISCUSSION

Brenner tumors are usually benign tumors, although there is a wide spectrum between benign and malignant features^(7)^. In contrast to the benign tumors, malignant Brenner tumor cells have hyperchromatic, pleomorphic nuclei and numerous mitotic figures, and they are characterized by destructive stromal invasion^(8,9)^. In our case series, 20 (90.9%) patients had benign Brenner tumors and two (9.1%) patients had malignant Brenner tumors (stage III C). Approximately 1% of Brenner tumors in the literature were determined as malignant^(3,10)^. Our results are inconsistent with the literature, but this might be because our clinic is a tertiary referral oncology center and the proportion of malignancy is higher than in the normal population.

Brenner tumors have a predilection for postmenopausal women in the literature^(3,11,12)^. In our study, the mean age of the patients was 55.1 years and 68.2% of the patients were postmenopausal, in accordance with the literature. Four patients (18.1%) had a mucinous cystadenoma. Coexistence of mucinous cystadenoma and Brenner tumor is consistent with the literature^(13)^. It is important that 25% of all mucinous ovarian tumors have a minor Brenner tumor component^(14)^. In our study, seven (33.3%) patients had postmenopausal bleeding and the final pathology of five patients was reported as endometrial carcinoma. Also, three patients presented with abnormal vaginal bleeding. In one previous study, the authors reported that histologically confirmed endometrial hyperplasia coexisted in 4-14% of women who had Brenner tumors^(15)^. Additionally, several cases were reported as having abnormal vaginal bleeding and endometrial hyperplasia in another study^(16)^. Synchronous multiple primary tumors of the female genital tract account for only 1-6% of all genital neoplasms. In the literature, the coexistence of endometrial cancer and ovarian cancer is the most frequently observed synchronous tumor occurrence, like in our study^(17,18)^. There are no data about the coexistence of cervical cancer and Brenner tumors in the literature; one (4.5%) patient had a grade 2 cervical cancer in our study.

Diagnosing Brenner tumors with imaging studies is difficult because the tumor’s appearance is nonspecific^(19,20)^. Therefore, before surgery, we could not estimate that Brenner tumors were present for any of the patients in our study. Although Brenner tumors are usually discovered incidentally, they sometimes have symptoms such as a palpable mass or pain^(6)^. Two patients presented with abdominal pain due to a Brenner tumor and there was no other pathologic finding for pain. These two cases were reported as malignant Brenner tumors and no benign Brenner tumors were symptomatic at the time of diagnosis.

Usually, benign Brenner tumors are unilateral and malignant Brenner tumors are bilateral^(21)^. In our study, one malignant tumor was bilateral and the other was unilateral. There was a benign Brenner tumor contralaterally in the unilateral malignant tumor case. All benign Brenner tumors were unilateral. Dierickx et al.^(20)^ and Blaustein’s^(8)^ textbook of pathology reported that prevelance of unilateral lesion was higher in the left ovary^(9)^. In our study, unilateral Brenner tumors were more common in left ovaries, in accordance with the literature.

Some authors indicated that malignant Brenner tumors had better prognosis than other epithelial ovarian tumors^(7)^. In our cases, there was no recurrence but this could be explained by the short follow-up period and the small number of malignant tumors.

The treatment for Brenner tumor is essentially surgical. Surgical staging should be done if a tumor has malignant potential. The role of lymphadenectomy is not yet clear because of the rare occurence of malignant Brenner tumors. It is reported that the rate of lymph node metastasis was 5.1% and lymphadenectomy was not associated with any improvement in survival^(22)^. Nasioudis et al.^(22)^ and Han et al.^(23)^ reported that the majority of malignant Brenner tumors presented with localized disease (stage I). In contrast to these published articles, the two patients with malignant tumors in our study presented at stage III, consistent with Gezginç et al.^(24)^.

### Study Limitations

In this study, data were retrieved retrospectively, which is the limitation of our study.

## CONCLUSION

Brenner tumors are usually benign neoplasms of the ovary, which are frequently diagnosed incidentally during surgical procedures. They are mostly seen in the postmenopausal period with vaginal bleeding and can coexist with other ovarian pathologies and female genital tumors.

Brenner tumors require further intervention trials and studies because of the limited number of trials.

## Figures and Tables

**Table 1:**
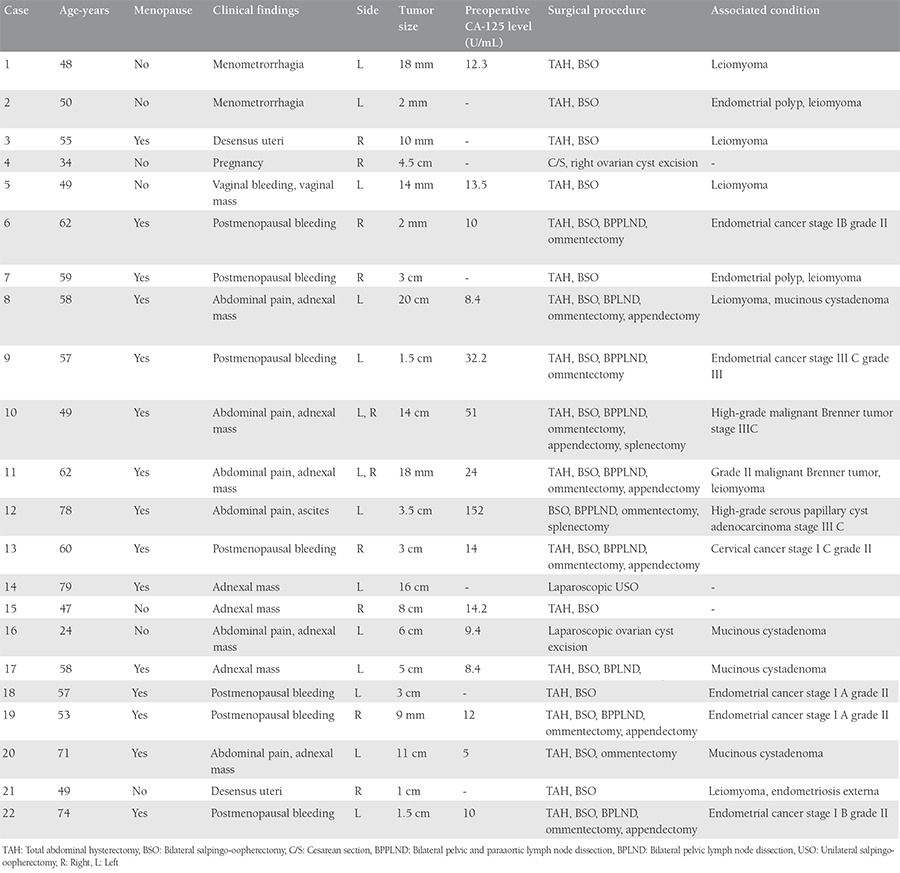
Clinical characteristics of the patients

## References

[1] Yamamoto R, Fujita M, Kuwabara M, Sogame M, Ebina Y, Sakuragi N, et al (1999). Malignant Brenner Tumors of the Ovary and Tumor Markers: Case Reports. Jpn J Clin Oncol.

[2] Balasa RW, Adcock LL, Prem KA, Dehner LP (1977). The Brenner tumor: a clinicopathologic review. Obstet Gynecol.

[3] Silverberg SG (1971). Brenner tumor of the ovary: a clinicopathologic study of 60 tumors in 54 women. Cancer.

[4] Jorgensen EO, Dockerty MB, Wilson RB, Welch JS (1970). Clinicopathologic study of 53 cases of Brenner’s tumors of the ovary. Am J Obstet Gynecol.

[5] Yoonessi M, Abell MR (1979). Brenner tumors of the ovary. Obstet Gynecol.

[6] Jung SE, Lee JM, Rha SE, Byun JY, Jung JI, Hahn ST (2002). CT and MRI imaging of ovarian tumors with emphasis on differential diagnosis. Radiographics.

[7] Roth LM, Gersell DJ, Ulbright TM (1993). Ovarian Brenner tumors and transitional cell carcinoma: recent developments. Int J Gynecol Pathol.

[8] Blaustein A (1982). Brenner tumors. In Pathology of the female genital tract (2nd edn). Springer-Verlag. New York.

[9] Meinhold-Heerlein I, Fotopoulou C, Harter P, Kurzeder C, Mustea A, Wimberger P, et al (2016). The new WHO classification of ovarian, fallopian tube, and primary peritoneal cancer and its clinical implications. Arch Gynecol Obstet.

[10] Westhuizen NG, Tiltman AJ (1988). Brenner tumours-a clinicopathological study. S Afr Med J.

[11] Green GE, Mortele KJ, Glickman JN, Benson CB (2006). Brenner tumors of the ovary: sonographic and computed tomographic imaging features. J Ultrasound Med.

[12] Athey PA, Siegel MF (1987). Sonographic features of Brenner tumor of the ovary. J Ultrasound Med.

[13] Nomura K, Aizawa S (1997). A histogenetic consideration of ovarian mucinous tumors based on an analysis of lesions associated with teratomas or Brenner tumors. Pathol Int.

[14] Scully RE, Young RH, Clement PB (1998) Tumors of the ovary, Maldeveloped Gonads, Fallopian Tube and Broad Ligaments. Armed Forces Institute of Pathology. Washington.

[15] Seldenrijk CA, Willig AP, Baak JP, Kühnel R, Rao BR, Burger CW, et al (1986). Malignant Brenner tumor. A histologic, morphometrical, immunohistochemical, and ultrastructural study. Cancer.

[16] Fox HA, Agrawal K, Langley FA (1972). The Brenner tumor of the ovary. A clinicopathological study of 54 cases. J Obstet Gynaecol Br Commonw.

[17] Matlock DL, Salem FA, Charles EH, Save EW (1982). Synchronous multiple primary neoplasms of the upper female genital tract. Gynecol Oncol.

[18] Schoenberg BS, Greenberg RA, Eisenberg H (1969). Occurrence of certain multiple primary cancers in females. J Natl Cancer Inst.

[19] Moon WJ, Koh BH, Kim SK, Kim YS, Rhim HC, Cho OK, et al (2000). Brenner tumor of the ovary: CT and MR findings. J Comput Assist Tomogr.

[20] Dierickx I, Valentin L, Van Holsbeke C, Jacomen G, Lissoni AA, Licameli A, et al (2012). Imaging in gynecological disease (7): clinical and ultrasound features of Brenner tumors of the ovary. Ultrasound Obstet Gynecol.

[21] Hermanns B, Faridi A, Rath W, Füzesi L, Schröder W (2000). Differential diagnosis, prognostic factors, and clinical treatment of proliferative Brenner tumor of the ovary. Ultrastruct Pathol.

[22] Nasioudis D, Sisti G, Holcomb K, Kanninen T, Witkin SS (2016). Malignant Brenner tumors of the ovary; a population-based analysis. Gynecol Oncol.

[23] Han JH, Kim DY, Lee SW, Park JY, Kim JH, Kim YM, et al (2015). Intensive systemic chemotherapy is effective against recurrent malignant Brenner tumor of the ovary: An analysis of 10 cases within a single center. Taiwan J Obstet Gynecol.

[24] Gezginç K, Karatayli R, Yazici F, Acar A, Çelik Ç, Çapar M, Tavli L (2012). Malignant Brenner tumor of the ovary: analysis of 13 cases. Int J Clin Oncol.

